# Expression of CD11b (MAC-1) and CD162 (PSGL-1) on monocytes is decreased under conditions of deep hypothermic circulatory arrest

**DOI:** 10.3892/etm.2014.1737

**Published:** 2014-05-28

**Authors:** STEFANIE SWOBODA, JOACHIM GRUETTNER, SIEGFRIED LANG, HANS-PETER WENDEL, MARTIN E. BEYER, EVA GRIESEL, HANS-MARTIN HOFFMEISTER, THOMAS WALTER

**Affiliations:** 1 Pharmacy Department of the University Hospital of Heidelberg, Heidelberg, Germany; 2 Emergency Department, University Medical Center Mannheim, Medical Faculty Mannheim, Heidelberg University, Mannheim, Germany; 3 First Department of Medicine (Cardiology), University Medical Center Mannheim, Medical Faculty Mannheim, Heidelberg University, Mannheim, Germany; 4 Clinic for Thoracic, Cardiac and Vascular Surgery, University of Tuebingen, Tuebingen, Germany; 5 Department of Internal Medicine II, Kirchheim Hospital, Kirchheim, Germany; 6 Department of Internal Medicine II, Solingen Hospital, Solingen, Germany

**Keywords:** CD11b, CD162, CD54, deep hypothermic circulatory arrest, leukocytes

## Abstract

Deep hypothermic circulatory arrest (DHCA) is a common technique used to protect vital organs during surgical interventions on the thoracic aorta or during surgery for complex congenital heart disease. Activated leukocytes are key mediators of inflammatory responses during ischemia. Intercellular crosstalk between leukocytes, platelets and endothelial cells is mediated by cell adhesion molecules. These molecules trigger complex cell-cell interaction mechanisms and initiate the release of proinflammatory molecules. One parameter that is known to have a significant impact on inflammatory cell activation and the production of proinflammatory markers is temperature. However, to the best of our knowledge, no data have yet been published on the effect of hypothermia on leukocyte surface markers during DHCA. Thus, the aim of the present study was to investigate the effect of hypothermia on the expression of cell adhesion molecules on monocytes under DHCA conditions *in vitro*. Blood samples collected from 11 healthy volunteers were incubated in a well-established model simulating circulatory arrest at 36°C and 18°C for 30 min. The expression of cluster of differentiation (CD) molecule 11B (CD11b), CD54 and CD162 on monocytes was measured as the mean fluorescence intensity (MFI) using flow cytometry. The expression level of CD11b on monocytes was significantly decreased following the incubation of the blood samples at 18°C compared with the level in blood samples incubated at 36°C (P<0.001). After 30 min of blood stasis in the circulatory arrest model, the expression level of CD162 on monocytes was significantly lower in the blood samples incubated at 18°C than in those incubated at 36°C (P<0.001). No association was identified between temperature and the surface expression of CD54 on monocytes following 30 min of stasis. These findings demonstrate that deep hypothermia decreases the expression of CD11b and CD162 on monocytes in an experimental setup simulating the conditions of DHCA. This may be the result of the inhibition of leukocyte-endothelial and leukocyte-platelet interactions, which may be a beneficial aspect of deep hypothermia that affects the inflammatory response and tissue damage during DHCA.

## Introduction

Deep hypothermic circulatory arrest (DHCA) has become a common technique used to protect vital organs during surgical interventions on the thoracic aorta in adults, neonates and infants undergoing surgery for complex congenital heart disease (1,2). Despite the excellent surgical results that may be obtained with DHCA, neurological dysfunction, renal failure and other organ damage remain common complications following DHCA (1,3). Bleeding also remains a constant risk following DHCA (3). An understanding of the complex interactions between various cells, such as leukocytes, platelets and endothelial cells, and the coagulation, fibrinolytic and inflammatory pathways, in addition to the impact of hypothermia on these pathways is essential for further improvement of DHCA.

One parameter that is known to have a significant impact on inflammatory cell activation is temperature (4). Hypothermia significantly decreases the global cerebral metabolic rate of glucose and oxygen. The metabolic rate of the human body at 18°C is only 12–25% of the metabolic rate at normal temperature (2). Experimental data has demonstrated that moderate hypothermia delays the production of proinflammatory cytokines and nuclear factor-κB (NF-κB) activation. NF-κB plays a pivotal role in regulating the transcription of cytokines, cell adhesion molecules and other mediators involved in the inflammatory response (5). Hypothermia may affect many metabolic pathways, reactions of inflammation and apoptosis processes, among many other pathways. A possible explanation for the success of therapeutic hypothermia is the multiple active mechanisms blocking the cascade of ischemia at a number of levels (6). In addition, hypothermia also affects hemostasis at various levels. It alters platelet function, the enzymatic kinetics in the coagulation cascade and the dynamic equilibrium of the fibrinolytic system (5).

Activated leukocytes are key mediators of inflammatory reactions due to their ability to release tissue-damaging compounds following their adherence to endothelial cells (7). Inflammatory cells, such as monocytes, are recruited in response to injury cues. They express a panoply of proinflammatory genes through a combination of transcription factors of which NF-κB is the most fundamental (8). The firm adhesion of leukocytes to the endothelium and their subsequent transmigration through the endothelium junctions represents the early stages of the inflammatory response following conditions such as ischemic injury, atherosclerosis and various other inflammatory disorders (9). Monocytes play an important role in regulating the thrombotic and fibrinolytic systems, and cell adhesion molecules trigger cellular interactions at the interface of thrombosis and fibrinolysis (10). Additionally, leukocyte-platelet interactions play an important role in inflammatory reactions. Both platelets and leukocytes are able to modulate each other’s functions. Leukocytes enhance platelet-mediated aggregation via interaction of their P-selectin ligand with P-selectin; binding of leukocytes to platelets promotes leukocyte activation (1,11). The cell-cell interaction between leukocytes, endothelial cells and platelets is mediated by adhesion molecules on the surface of these cells. Thus, modulation of the expression of cell adhesion molecules on leukocytes during blood stasis and hypothermia may have an impact on inflammatory responses during DHCA.

Studies concerning hypothermic conditions have mainly focused on platelet activation, platelet-leukocyte interactions or inflammatory markers; however, to the best of our knowledge, no data are available concerning the expression of cell adhesion molecules in leukocytes during DHCA. The purpose of the present study was to investigate the expression of the cell adhesion molecules cluster of differentiation (CD) molecule 11B [CD11b; also known as α subunit of the β2-integrin macrophage-1 antigen (MAC-1)], CD54 [also known as intercellular adhesion molecule 1 (ICAM-1)] and CD162 [also known as P-selectin glycoprotein ligand 1 (PSGL-1)] on monocytes under normothermic (36°C) and hypothermic (18°C) conditions using an *in vitro* experimental model that simulates the conditions of circulatory arrest.

## Materials and methods

### Subjects

Healthy volunteers were included in the present study following the provision of informed consent. The study protocol conforms to the ethical guidelines of the 1975 Declaration of Helsinki and was approved by the local Ethics Committee (University of Tuebingen, Tuebingen, Germany).

### Blood sampling and sample preparation

Blood from non-medicated healthy male volunteers (n=11) was collected by venipuncture with a 21-gauge needle from an antecubital vein. The first 5 ml of blood was discharged prior to the drawing of additional blood samples for analysis. All blood samples were anti-coagulated with 3 U/ml heparin.

One blood sample set from each subject was incubated in stasis for 30 min at 18°C to simulate DHCA. The other blood sample set was incubated in stasis for 30 min at 36°C. Each of the sample sets (18°C and 36°C) consisted of three subsets: i) one subset was incubated with an antibody combination of anti-CD45 and anti-CD11b; ii) the second subset was incubated with anti-CD45 and anti-CD54; and iii) the third subset was incubated with anti-CD45 and anti-CD162, as described below. For incubation of the blood samples, a specially designed temperature-regulation device with a heating and cooling function was used (11). The model was intended to mimic the conditions of stasis and hypothermia.

### Sub-samples preparation for flow-cytometry

The following incubation steps were performed immediately after the 30 min period of incubation in stasis using a previously described method (12,13).

Expression of the following cell adhesion molecules on monocytes was measured by flow cytometry (Coulter Epics XL-MCL™; Beckman Coulter, Krefeld, Germany). Leukocytes were detected using fluorescein isothiocyanate (FITC)-conjugated anti-lymphocyte common antigen (anti-CD45; BD Biosciences, Heidelberg, Germany). The following phycoerythrin (PE)-conjugated monoclonal antibodies were used for cell detection in fluorescence-activated cell sorting (FACS): Anti-CD11b, anti-CD54 (both from BD Biosciences) and anti-CD162 (Beckman Coulter). Whole blood (100 μl) was incubated with saturating concentrations of FITC-conjugated anti-CD45 and PE-conjugated monoclonal antibodies for 20 min at room temperature. Erythrocytes were lysed and leukocytes were fixed with a commercially available solution (FACS Lysing Solution, BD Biosciences). Samples were then incubated for 10 min in the dark. Thereafter, samples were centrifuged at 200 × g for 10 min, the pellet washed with phosphate-buffered saline (Gibco, Invitrogen Life Technologies, Karlsruhe, Germany) and centrifuged again. The pellet was then resuspended in phosphate-buffered saline and applied to the flow cytometer equipped with a 488 nm argon laser. Results are expressed as mean fluorescence intensity (MFI) of CD11b, CD54 and CD162 on monocytes.

### Statistical methods

The Mann-Whitney U test for independent samples was used to analyze the significance of any differences between samples incubated at 18°C and samples incubated at 36°C. Data are presented as median and interquartile range (IQR, 25th to 75th percentiles). A two-tailed P value <0.05 was considered to indicate a statistically significant difference. The calculations were performed using InStat (GraphPad Software, Inc., San Diego, CA, USA) and IBM SPSS software (IBM, Ehningen, Germany).

## Results

The volunteers (n=11) had no history of acute or chronic disease and were non-medicated. Their mean age was 28.7 years. All donors were male (ethnic background: all Caucasian).

Following an incubation period of 30 min in a circulatory arrest model simulating blood stasis with different temperatures, a significant association between temperature and surface expression of CD11b and CD162 on monocytes was demonstrated.

**Figure 1:**
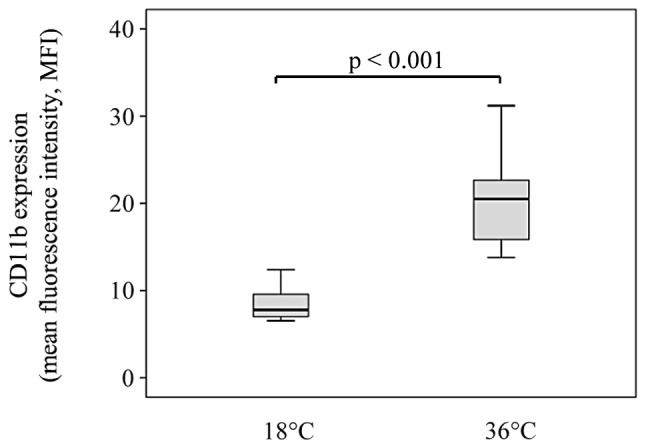
Expression of cluster of differentiation 11B (CD11b; macrophage-1 antigen; MAC-1) on monocytes at normothermia and hypothermia. Data are presented as medians of mean fluorescence intensity with 25th and 75th percentiles (boxes) and 10th and 90th percentiles (whiskers).

**Figure 2:**
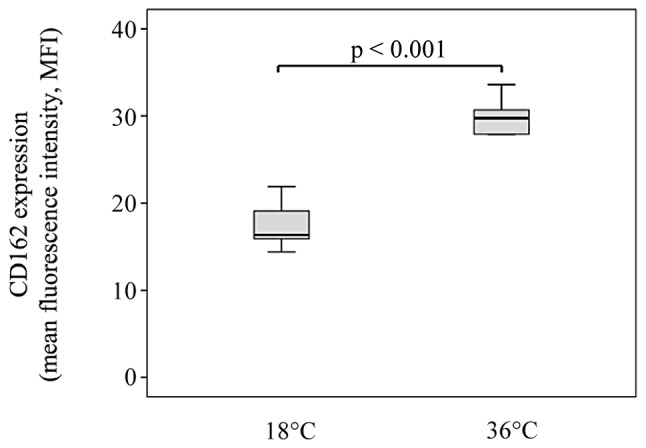
Expression of cluster of differentiation 162 (CD162; P-selectin glycoprotein ligand-1; PSGL-1) on monocytes at normothermia and hypothermia. Data are presented as medians of mean fluorescence intensity with 25th and 75th percentiles (boxes) and 10th and 90th percentiles (whiskers).

**Figure 3:**
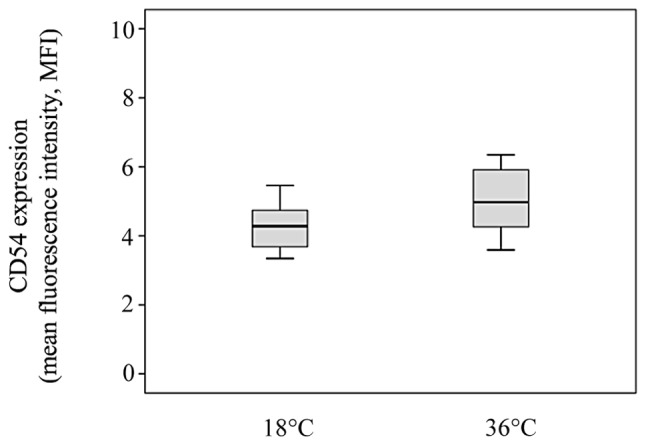
Expression of cluster of differentiation 54 (CD54; intercellular adhesion molecule 1; ICAM-1) on monocytes at normothermia and hypothermia. Data are presented as medians of mean fluorescence intensity with 25th and 75th percentiles (boxes) and 10th and 90th percentiles (whiskers).

The expression of CD11b on monocytes was significantly decreased following the incubation of blood at 18°C compared with the expression of CD11b on monocytes after incubation at 36°C (median MFI 7.78, IQR 7.0–9.6 vs. median MFI 20.5, IQR 15.4–22.7, respectively, P<0.001). After 30 min of blood stasis in the circulatory arrest model, the expression of CD162 on monocytes was significantly lower at 18°C compared with that at 36°C (median MFI 16.4, IQR 15.9–19.1 vs. median MFI 29.8, IQR 27.9–30.7, respectively, P<0.001). No association was identified between temperature and the surface expression of CD54 on monocytes after 30 min of stasis. The expression of CD54 on monocytes did not differ between 18°C and 36°C (median MFI 4.28, IQR 3.7–4.7 vs. median MFI 4.97, IQR 4.3–6.0, respectively, P>0.05). Medians with quartiles for MFI are depicted in Figs. 1–3.

## Discussion

During certain cardiac surgical procedures, such as those on the thoracic aorta or during the repair of congenital cardiac defects, extracorporeal circulation (ECC) in combination with deep hypothermia (<20°C) may be performed, particularly during deep hypothermic circulatory arrest (DHCA) (14). DHCA has been demonstrated to be an effective and safe organ protection technique used during periods of interrupted blood flow (2,15). The physiological basis for the preservation of tissue integrity during a period of interrupted circulation through hypothermia centers on the lowering of the metabolic rate through a reduction in temperature (15). Another reason is a reduced systemic inflammatory response with DHCA since temperature has a significant impact on inflammatory cell activation (4,16). Activated leukocytes are key mediators of inflammatory reactions due to their ability to release tissue-damaging compounds following their adherence to endothelial cells (7). Hypothermia is known to have anti-inflammatory effects by inhibiting leukocyte response following several types tissue insults such as ischemic brain or liver injury (4). The interaction between leukocytes, endothelial cells and platelets is mediated by cell adhesion molecules that are expressed on the surface of activated cells. In a prior investigation, a temperature-dependent regulation of the surface expression of CD11b and CD162 on monocytes in an ECC model was demonstrated (13). However, to the best of our knowledge, no data are available to date regarding the expression of cell adhesion molecules on leukocytes during DHCA.

In the present *in vitro* study, the effects of blood temperature on the surface expression of cell adhesion molecules on monocytes were investigated using an experimental model simulating circulatory arrest. A difference in the expression levels of CD11b and CD162 on monocytes was observed between blood incubated at 18°C for 30 min compared with blood incubated at 36°C for the same time period.

CD11b/CD18 (MAC-1) is a member of the β2-integrin family. It is present in an inactive state on circulating leukocytes; however, upon neutrophil stimulation with various cytokines it undergoes a rapid conformational change that results in its activation, which is required for optimal integrin function. β2-integrins mediate leukocyte adhesion and transmigration across the endothelium, through interactions with ICAM-1 on the activated endothelium. Several adhesion-dependent neutrophil functions, such as binding to fibrinogen, immune complexes and platelets, are MAC-1-dependent owing to the large variety of ligands for MAC-1 and its ability to cooperate functionally with a variety of other surface receptors (17). The P-selectin/MAC-1 cascade gives rise to heterotypic conjugates of platelets with leukocytes. Leukocyte tethering by the P-selectin of platelets not only induces rapid β2-integrin activation but also triggers delayed responses through the induction of the expression of transcription factors such as NF-κB. This activates gene transcription for the synthesis of proinflammatory molecules; these are fundamental for leukocytes to acquire an inflammatory phenotype (18). Experimental data demonstrate that hypothermia delays the production of proinflammatory cytokines and NF-κB activation (5). Furthermore, studies on platelet function have reported a DHCA-induced platelet dysfunction, and hypothermia has been revealed to induce platelet-aggregate formation (1,3). Additionally, soluble fibrinogen promotes neutrophil activation in a MAC-1-dependent manner and the interaction of platelets with leukocytes may result in fibrin deposition through increased tissue-factor expression (17,18). Tissue-type plasminogen activator (tPA) promotes the aggregation and interaction of annexin A2 and MAC-1, leading to the clustering and activation of MAC-1 signaling in macrophages (8). Thus, CD11b (MAC-1) is a surface receptor on stimulated monocytes and neutrophils. It is very important for cell-cell interactions which contribute to the inflammatory response and also contribute to hemostasis. Several clinical and experimental studies have suggested that the expression of integrins on leukocytes is upregulated during or after cardio-pulmonary bypass (CPB) (19–21). Experimental data have demonstrated that cooling decreases the upregulation of MAC-1 on monocytes (4,13). The use of hypothermia has been shown to significantly reduce the expression of MAC-1 during CPB compared with that during normothermia in cardiac surgical patients (7). In the present investigation the results revealed a significantly decreased expression of MAC-1 on monocytes during conditions of DHCA compared with that during normothermia. This suggests that hypothermia reduces the upregulation of CD11b (MAC-1) expression on monocytes during conditions of DHCA. This is consistent with data demonstrating that hypothermia delays the production of proinflammatory cytokines and NF-κB activation (5). Reduced expression of MAC-1 on monocytes during circulatory arrest may be associated with a decreased ability of the leukocytes to adhere to endothelium and platelets, which is a prerequisite for reduced leukocyte-induced tissue damage. Thus, hypothermia-induced reduction of CD11b expression on monocytes is part of the organ-protective effects during DHCA.

CD54 (ICAM-1) is a member of the immunoglobulin superfamily. It is expressed on the cell surface of a wide variety of cell types including endothelial cells and leukocytes, functioning as a key receptor in the cell-cell interactions (9,22). During inflammation, flowing leukocytes roll onto vascular surfaces, arrest, spread, crawl to endothelial junctions and then migrate into extravascular tissues. Selectin-ligand interactions initiate rolling, whereas integrin-ligand interactions mediate arrest and crawling. As the neutrophils roll, P- or E-selectin expressed on activated endothelial cells transduces signals that partially activate integrin CD11a, which binds reversibly to ICAM-1 to decrease the rolling velocities. Slow rolling facilitates neutrophil interactions with endothelial cell-bound chemokines that fully activate the integrins, leading to arrest (23). The binding of ICAM-1 to MAC-1 (CD11b/CD18) results in the adhesion of neutrophils and monocytes to the endothelium (22). ICAM-1 is induced by cytokines and various stress stimuli such as hypoxia, and is associated with a variety of inflammatory diseases and conditions, including atherosclerosis and ischemia reperfusion injury (9,22). Such inflammatory conditions are present during DHCA. The NF-κB signaling cascade is pivotal in ICAM-1 activation but NF-κB-independent pathways may also participate (9). The NF-κB signaling pathway is activated by the proinflammatory cytokines tumor necrosis factor-α (TNF-α) and interleukin-1β (IL-1β), the major inducers of ICAM-1 expression in most cell types (22). Experimental data demonstrate that hypothermia delays the production of the proinflammatory cytokines TNF-α and IL-1β as well as NF-κB activation (5). Thus, it may be speculated that hypothermia decreases the expression of ICAM-1 on the cell surface by attenuating the activation of proinflammatory cytokines and NF-κB. In contrast to this hypothesis, a recent investigation revealed no association between different blood temperatures and the expression of CD54 on monocytes after 30 min of blood circulation in an *in vitro* ECC model (13). In the present study, the effect of deep hypothermia during blood stasis using an experimental setup simulating DHCA was investigated. The data demonstrate no significant difference in the surface expression of ICAM-1 (CD54) on monocytes during normothermia and hypothermia. An explanation for this may be that alternative activation pathways of ICAM-1 exist via NF-κB-independent pathways (9,22). These NF-κB-independent signaling pathways are likely to be unaffected by hypothermia. Nevertheless, the data demonstrate that even though the expression of ICAM-1 on monocytes was unaffected by hypothermia during stasis, the leukocyte-endothelial interaction was reduced by deep hypothermia due to the decreased expression of its ligand MAC-1 on monocytes. Thus, deep hypothermia leads to an inhibited leukocyte response during circulatory arrest.

During acute inflammation, leukocytes are recruited from the blood circulation to sites of infection and injury. This multistep adhesion and signaling cascade is initiated by interactions between selectins and their glycoconjugates that mediate leukocyte tethering to and rolling on the surface of endothelial cells. CD162 (PSGL-1) has been demonstrated to generate a specific, high-affinity, biologically relevant ligand for P-selectin (24,25). To trigger slow leukocyte rolling on ICAM-1, neutrophils rolling on P-selectin engage PSGL-1 (23). In addition, ligation of PSGL-1 by P-selectin may trigger intracellular events in some leukocytes which enable them to respond to mediators elaborated at sites of inflammation (24,25). P-selectin is an adhesion molecule, which, besides generating a tether with PSGL-1, induces a signal that activates leukocytes through a molecular cascade, finally inducing the activated form of MAC-1. The P-selectin/MAC-1 cascade gives rise to heterotypic conjugates of platelets with leukocytes (18). It has been reported by Straub *et al*, that hypothermia induces α-granule release with increased expression of P-selectin on platelets, which mediates platelet-leukocyte binding via interaction with the leukocyte ligand PSGL-1. This finding reveals that hypothermia induces platelet activation (1,11). In a prior study, a decreased expression level of CD162 on monocytes at 18°C was revealed using an *in vitro* ECC model (13). In concordance with these results, the current study demonstrated a decreased expression of CD162 on monocytes at deep hypothermia during blood stasis. The results of the present study suggest that hypothermia decreases PSGL-1 expression on monocytes resulting in a decreased platelet-leukocyte interaction. Additionally, hypothermia-induced reduction of PSGL-1 on monocytes may further have an impact on leukocyte-endothelial interaction, such as the tethering and rolling of leukocytes, which affects the inflammatory response. These data suggest that deep hypothermia has a beneficial effect on the systemic inflammatory response and organ damage during circulatory arrest.

In conclusion, to the best of our knowledge, the findings of the present study reveal for the first time that deep hypothermia decreases the expression of CD11b (MAC-1) and CD162 (PSGL-1) on monocytes in an experimental setup simulating the conditions of DHCA. This may result in an inhibition of leukocyte-endothelial and leukocyte-platelet interactions, which may be a beneficial aspect of deep hypothermia that may influence the inflammatory response and tissue damage during DHCA.
